# Quantification of diacylglycerol and triacylglycerol species in human fecal samples by flow injection Fourier transform mass spectrometry

**DOI:** 10.1007/s00216-020-02416-y

**Published:** 2020-03-21

**Authors:** Verena M. Ertl, Marcus Höring, Hans-Frieder Schött, Christina Blücher, Louise Kjølbæk, Arne Astrup, Ralph Burkhardt, Gerhard Liebisch

**Affiliations:** 1 Institute of Clinical Chemistry and Laboratory Medicine, University Hospital Regensburg, Franz-Josef-Strauß-Allee 11, 93053 Regensburg, Germany; 2 Department of Nutrition, Exercise and Sports, Faculty of Science, University of Copenhagen, Nørre Allé 51, 2200 Copenhagen, Denmark

**Keywords:** Lipidomics, Microbiome, Feces, Triglyceride, Diglyceride, High-resolution mass spectrometry

## Abstract

The intestinal microbiome plays an important role in human health and disease and fecal materials reflect the microbial activity. Thus, analysis of fecal metabolites provides insight in metabolic interactions between gut microbiota and host organism. In this work, we applied flow injection analysis coupled to Fourier transform mass spectrometry (FIA-FTMS) to identify and quantify lipid species in human fecal samples. Fecal homogenates were subjected to lipid extraction and analyzed by FIA-FTMS. The analysis of different subjects revealed a vast heterogeneity of lipid species abundance. The majority of samples displayed prominent signals of triacylglycerol (TG) and diacylglycerol (DG) species that could be verified by MS2 spectra. Therefore, we focused on the quantification of TG and DG. Method validation included limit of quantification, linearity, evaluation of matrix effects, recovery, and reproducibility. The validation experiments demonstrated the suitability of the method, with exception for approximately 10% of samples, where we observed coefficients of variation higher than 15%. Impaired reproducibility was related to sample inhomogeneity and could not be improved by additional sample preparation steps. Additionally, these experiments demonstrated that compared with aqueous samples, samples containing isopropanol showed higher amounts of DG, presumably due to lysis of bacteria and increased TG lipolysis. These effects were sample-specific and substantiate the high heterogeneity of fecal materials as well as the need for further evaluation of pre-analytic conditions. In summary, FIA-FTMS offers a fast and accurate tool to quantify DG and TG species and is suitable to provide insight into the fecal lipidome and its role in health and disease.

**Electronic supplementary material:**

The online version of this article (10.1007/s00216-020-02416-y) contains supplementary material, which is available to authorized users.

## Introduction

It is now generally accepted that the gastrointestinal system in particular the intestinal microbiome plays an important role in human health and disease [1]. Microbial activity is reflected in fecal materials that contain unabsorbed metabolites including lipid species. Consequently, analysis of fecal metabolites provides an estimate of metabolic interaction between gut microbiota and host [2]. To identify subtle metabolic variations induced by dietary alterations and to characterize the metabolic impact of variations of the gut microbiota, metabolic profiling gained increasing interest over the last decade.

Feces are composed of water, proteins, bacterial biomass, fat, and indigestible food components, e.g., fibers. Fat contained in feces is a heterogeneous mixture of different lipids and constitutes 8–16% of the dry weight of feces [3–5] and 2–8% of wet weight [6–10]. Fat found within feces comes from bacteria as well as from the undigested remains of dietary lipids [11]. Approximately 60–70% represents non-/esterified fatty acids; 20–30% is unsaponifiable material [12]. Human feces contain, depending on diet and metabolism, different amounts of triacylglycerol (TG) and diacylglycerol (DG), which has been frequently studied in the context of steatorrhea [13] and colon cancer [14].

Lipidomic methods nowadays offer a wide range of possibilities to analyze lipid species profiles of biological materials [15]. However, only a few methods are available to study the lipidome of fecal material [2, 16, 17]. Most of the described approaches focus on the identification and quantification of selected lipid classes like fatty acids [18, 19], bile acids [20], and sterols [21]. Here, we report the evaluation and validation of a method for identification and quantification of DG and TG species of human fecal material using flow injection analysis (FIA) coupled to Fourier transform mass spectrometry (FIA-FTMS).

## Materials and methods

### Chemicals and reagents

Methanol and ethanol absolute (EMSURE) were obtained from Merck (Darmstadt, Germany), and chloroform and 2-propanol from Roth (Karlsruhe, Germany). All solvents were of HPLC grade. Ammonium formate was ordered from Sigma-Aldrich (Taufkirchen, Germany) and isooctane (2,2,4-trimethylpentane) > 99% from Honeywell (Seelze, Germany). All chemicals and standards were of high purity grade for analysis (> 95%). Glycerolipid standards were purchased from Larodan (Solna, Sweden): diarachidin (DG 20:0/20:0), dinonadecanoin (DG 19:0/19:0), dilinolenin (DG 18:3/18:3), dilinolein (DG 18:2/18:2), 1,2-distearin (DG 18:0/18:0), triarachidin (TG 20:0/20:0/20:0), trinonadecanoin (TG 19:0/19:0/19:0), trilinolein (TG 18:2/18:2/18:2), triolein (TG 18:1/18:1/18:1), 1,2-olein-3-stearin (TG 18:1/18:1/18:0), 1,2-stearin-3-olein (TG 18:0/18:0/18:1), triheptadecanoin (TG 17:0/17:0/17:0), and tripalmitin (TG 16:0/16:0/16:0). Purified water was produced by Millipore Milli-Q UF-Plus water purification system (Molsheim, France).

### Stock solutions

All diacylglycerol and triacylglycerol standards were dissolved in isooctane/isopropanol (3:1 v/v) with a concentration of 1.0 mg/mL. The internal standard (IS) solution contained trinonadecanoin, triheptadecanoin, and diarachidin each at a concentration of 10 μg/mL in chloroform/methanol (9:1 v/v).

### Samples

Human fecal material was obtained from 20 healthy volunteers for method development. The material was collected in the morning and directly transported to the laboratory (stored on ice). Polypropylene tubes were used for sample collection, immediately stored at − 20 °C, and transported to the laboratory on ice. Samples were stored at − 80 °C until further processing. Samples used to investigate the influence of stool grade were collected as described by Kjølbæk et al. [22]. This trial was registered under ClinicalTrials.gov Identifier no. NCT02215343.

### Sample preparation

A randomly selected part of the raw fecal material was homogenized in isopropanol/water (70/30, v/v) using a gentleMACS^™^ Dissociator (Miltenyi Biotec GmbH, Bergisch Gladbach, Germany) as described previously [21]. The homogenate was diluted in 70% isopropanol to a concentration of 2.0 mg dry weight/mL (dw/mL) for further analysis. Samples were always kept on ice and stored at − 80 °C until further processing. An amount of 50 μL of the internal standard solution (containing 0.54 nmol TG 57:0, 0.59 nmol TG 51:0, and 0.73 nmol DG 40:0) was added to a sample volume of 100 μL (2 mg dw/mL) fecal homogenate prior to lipid extraction and extracted according to the protocol of Bligh and Dyer [23] with a total chloroform volume of 2 mL and an extraction time of 60 min at room temperature. A volume of 1200 μL of the separated chloroform phase was transferred into a sample vial by a pipetting robot (Tecan Genesis RSP 150) and evaporated to dryness in a vacuum concentrator. The residues were dissolved in 1.0 mL chloroform/methanol/2-propanol (1:2:4 v/v/v) containing 7.5 mM ammonium formate.

### Flow injection Fourier transform mass spectrometry

Mass spectrometric analysis of the reconstituted lipid extracts was performed by direct flow injection analysis using Fourier transform mass spectrometry (FIA-FTMS). A hybrid quadrupole-Orbitrap mass spectrometer (QExactive, Thermo Fisher Scientific, Bremen, Germany) equipped with a heated electrospray ionization source was coupled to a PAL autosampler (CTC Analytics, Zwingen, Switzerland) and an UltiMate 3000 isocratic pump (Thermo Fisher Scientific, Waltham, MA, USA). The injection volume was 50 μL and a solvent mixture of chloroform/methanol/2-propanol (1:2:4 v/v/v) delivered at an initial flow rate of 100 μL/min until 0.25 min, followed by 10 μL/min for 2.5 min and a washout with 300 μL/min for 0.5 min. The ion source was operated in positive ion mode using the following parameters: spray voltage 3.5 kV, capillary temperature of 281 °C, S-lens RF level 55, aux gas heater temperature of 250 °C, and flow rates of 58 for sheath gas and 16 for aux gas. FTMS data were recorded in positive ion mode with a maximum injection time (IT) of 200 ms, an automated gain control (AGC) of 1·10^6^, three microscans, and a target resolution of 140,000 (at *m/z* 200). Diacylglycerols were measured in a mass rage *m/z* 450–800 and triacylglycerols in a range of *m/z* 750–1200. MS2 spectra were acquired for 3 min in mass range *m/z* 450–1200 with a step size of 1.0008 Da and an isolation window of 1 Da with a normalized collision energy of 20%, an IT of 64 ms, AGC of 1·10^5^, and a target resolution of 17,500.

### Lipid identification and data processing

ALEX software [24] was used for peak assignment of data acquired by FTMS and MS/FTMS (MS2) using an *m/z* tolerance of ± 0.0045 Da. Peaks with mass deviation of more than 3 ppm were not considered. Species assignment included evaluation of product ion spectra (see Electronic Supplementary Material (ESM) Fig. S2). The assigned data were exported to Microsoft Excel 2010 and processed using self-programmed macros. For accurate quantification, intensities were corrected for type I isotope effects (relative isotope abundance; [25]). Type II corrections (overlap mainly resulting from ^13^C-atoms) were not required at the selected mass resolution due to peak coalescence (Hoering et al., manuscript in preparation). Quantification was performed by normalization of analyte to internal standard intensities multiplied with the spiked amount of the internal standard as described recently [26]. Lipids were annotated as sum composition of acyl chains or without specification of *sn* positions using “_” as previously proposed [27].

### Method validation

Limit of quantification (LoQ) of DG and TG species was determined from serial dilutions of fecal samples. Each level was analyzed in fivefold. The coefficient of variation (CV) and the absolute value of trueness – 100% were determined and plotted against the concentrations. The results were fitted by a power function. LoQ was calculated representing a CV of ≤ 20% and absolute value of trueness – 100% ≤ 20%, respectively. The higher concentration of both calculations was defined as LoQ (for details, see ESM).

Intra-day precision was assessed for five different samples which were extracted five times and quantified. For inter-day precision, the same samples were extracted and measured on five different days (20 days between first and last measurements).

Linearity of quantification was determined using spiked samples at six concentration levels. Each level was extracted 5-fold. The results were fitted by a linear function.

Dilution integrity of DG and TG species was determined by analysis of stool samples at different concentrations (from 1.6 to 0.02 mg dw/mL). Samples were measured in triplicates. The measured quantity was compared with the target quantity determined at the highest sample concentration.

### Microscopy

The particle size was documented using phase-contrast microscopy with × 10 magnification (Zeiss Primovert, Jena, Germany) and the ZEN 2.6 lite imaging software.

## Results and discussion

**Fig. 1:**
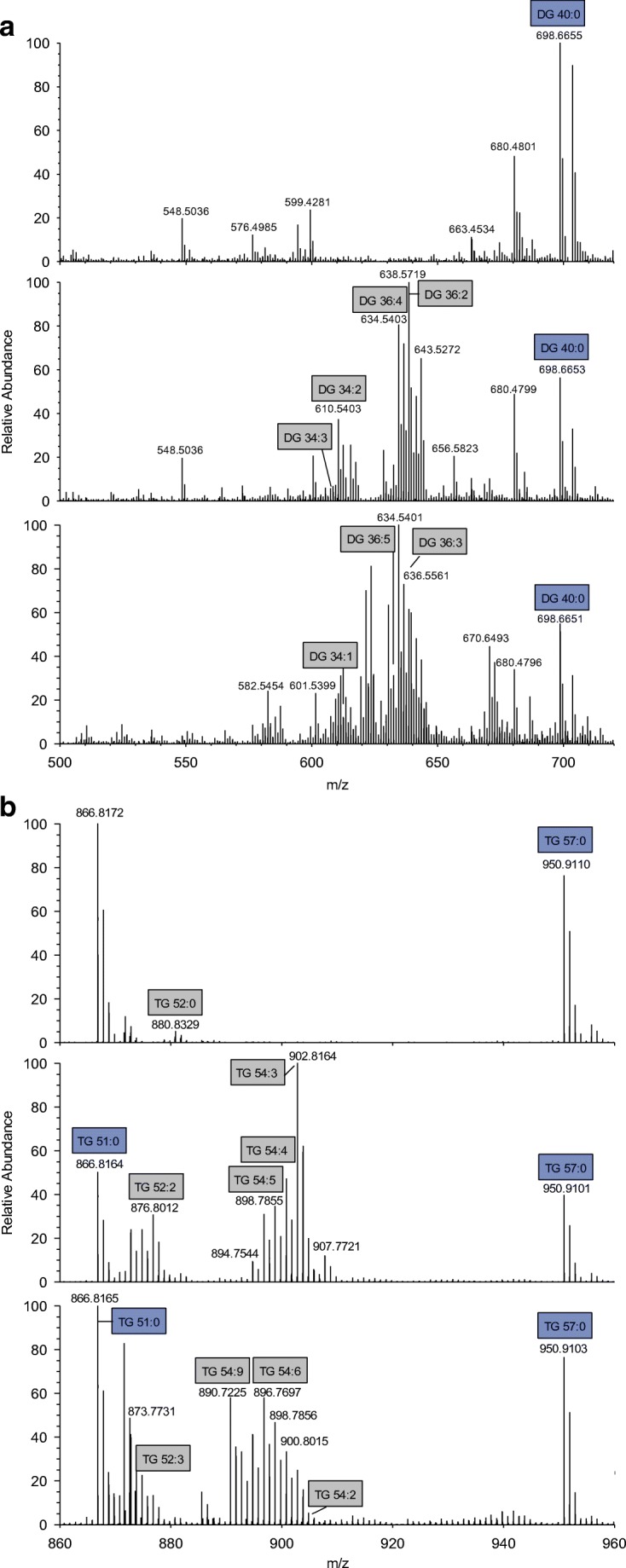
Displayed are mass spectra from three individual human fecal samples analyzed in positive ion mode. Panel **a** shows the mass range of DG species (*m/z* 500–720) and panel **b** of TG species (*m/z* 810–980)

Our initial aim was to develop an accurate and fast method for the identification and quantification of lipid species in human fecal material using FIA-FTMS with a quadrupole-Orbitrap hybrid mass spectrometer (QExactive). Crude lipid extracts prepared by chloroform extraction according to the protocol by Bligh and Dyer [23] were analyzed in positive ion mode. Upon initial evaluation, spectra revealed a high heterogeneity (Fig. 1) and numerous peaks could be assigned to [M+NH_4_]^+^ ions of DG and TG species. Other lipid classes were not detected in significant amounts not even in negative ion mode spectra (data not shown). Therefore, we decided to focus on the quantification of DG and TG species.

In a first step, 20 different fecal samples were screened for their DG and TG content. None of the analyzed samples contained signals representing a relevant interference with the selected internal standards (IS) DG 40:0, TG 51:0, and TG 57:0 (ESM Fig. S1). To prove the identity of detected species, MS2 spectra were evaluated and product ions assigned according to the annotation system proposed recently [28] (exemplified in ESM Fig. S2). The concentrations of DG and TG species detected in these samples span a range up to or more than three orders of magnitude (Table 1). Highest mean concentrations were detected for polyunsaturated species with more than two double bonds: DG 36:3, DG 36:4, TG 54:3, TG 54:4, and TG 54:5. The detected acyl fragments comprised mainly acyl chains with 16 and 18 carbons and up to three double bonds. For DG, also species containing FA 12:0 and 14:0 were detected precluding application of DG 28:0 as IS.

**Table 1:** Concentrations and acyl combinations of DG and TG species in human feces from 20 different samples. Data based on a single measurement of the individual samples and acyl combinations were derived from MS2 spectra

Compound	[M+NH_4_]^+^*m/z*	Mean ± standard deviation (nmol/mg dw)*	Median	Min	Max	Acyl combinations
DG 26:0	502.447	0.139 ± 0.348	0.004	n.d.	1.401	DG 12:0_14:0
DG 28:0	530.478	0.080 ± 0.214	0.002	n.d.	0.919	DG 12:0_16:0
						DG 14:0_14:0
DG 30:0	558.509	0.106 ± 0.322	0.008	n.d.	1.454	DG 12:0_18:0
						DG 14:0_16:0
DG 34:3	608.525	0.428 ± 0.660	0.240	n.d.	2.808	DG 16:0_18:3
DG 34:2	610.541	1.865 ± 1.856	0.994	0.045	6.198	DG 16:0_18:2
DG 34:1	612.556	0.776 ± 0.711	0.503	0.068	2.417	DG 16:0_18:1
DG 36:5	632.525	1.254 ± 1.922	0.726	0.002	8.481	DG 18:2_18:3
DG 36:4	634.541	7.305 ± 8.349	4.302	0.053	32.355	DG 18:2_18:2
						DG 18:1_18:3
DG 36:3	636.556	4.371 ± 4.513	2.452	0.042	14.508	DG 18:1_18:2
DG 36:2	638.572	3.632 ± 4.283	2.564	0.074	17.947	DG 18:1_18:1
						DG 18:0_18:2
TG 48:0	824.770	0.042 ± 0.104	0.010	n.d.	0.491	TG 16:0_16:0_16:0
TG 50:3	846.755	0.475 ± 0.465	0.445	0.018	1.008	TG 16:0_16:1_18:2
TG 50:2	848.770	0.141 ± 0.253	0.072	0.003	1.150	TG 16:0_16:0_18:2
						TG 16:0_16:1_18:1
TG 50:1	850.786	0.083 ± 0.116	0.032	0.006	0.430	TG 16:0_16:0_18:1
TG 50:0	852.801	0.164 ± 0.466	0.031	n.d.	2.088	TG 16:0_16:0_18:0
TG 52:5	870.755	0.105 ± 0.143	0.045	n.d.	0.566	TG 16:1_18:2_18:2
						TG 16:0_18:2_18:3
TG 52:4	872.770	0.841 ± 1.284	0.494	n.d.	5.869	TG 16:0_18:2_18:2
						TG 16:1_18:1_18:1
TG 52:3	874.786	0.487 ± 0.901	0.174	n.d.	3.666	TG 16:0_18:1_18:2
TG 52:2	876.801	0.459 ± 0.892	0.127	0.009	3.465	TG 16:0_18:1_18:1
						TG 16:0_18:0_18:2
TG 53:4	886.786	0.269 ± 0.270	0.234	0.005	0.624	TG 17:1_18:1_18:2
TG 54:9	890.723	0.078 ± 0.160	0.003	n.d.	0.641	TG 18:3_18:3_18:3
TG 54:7	894.755	0.409 ± 0.528	0.080	n.d.	1.895	TG 18:2_18:2_18:3
TG 54:6	896.770	1.238 ± 1.721	0.684	n.d.	7.388	TG 18:0_18:3_18:3
						TG 18:1_18:2_18:3
						TG 18:2_18:2_18:2
TG 54:5	898.786	1.174 ± 1.886	0.599	0.003	7.875	TG 18:1_18:2_18:2
TG 54:4	900.801	1.175 ± 2.250	0.422	0.003	8.988	TG 18:1_18:1_18:2
						TG 18:0_18:1_18:3
TG 54:3	902.817	1.523 ± 3.239	0.294	0.008	11.560	TG 18:1_18:1_18:1
						TG 18:0_18:1_18:2

### Reproducibility

In an important next step within method development [29, 30], we evaluated the performance of the FIA-FTMS method. Due to sample heterogeneity, intra- and inter-day precisions were evaluated in five different samples (Tables 2 and 3). The coefficients of variation (CVs) were below 15% or even below 10% for most DG species. For sample 5 significantly higher variations were observed especially for TG species concentrations (see also “Evaluation of reproducibility issues”). Moreover, we observed for this sample a decrease in the concentrations of most of the TG species from day to day. Despite storage of the samples in 70% isopropanol at − 80 °C, this decline may be related to lipase activity since enzymatic activity has been reported also in organic solvents [31, 32].

**Table 2:** Coefficient of variation (CV) of intra- and inter-day precision of DG species determined in five different human fecal samples by FIA-FTMS/MS analyzed in fivefold

Diacylglycerols		Intra-day	CV (%)	Inter-day	CV (%)
	Sample	Mean (*n* = 5) (nmol/mg dw)		Mean (*n* = 5) (nmol/mg dw)	
DG 34:3	Sample 1	*0.13*	7.2	*0.13*	11.6
	Sample 2	*0.36*	16.0	*0.38*	13.7
	Sample 3	*0.61*	6.4	*0.63*	8.1
	Sample 4	*0.59*	3.7	*0.53*	4.3
	Sample 5	*0.12*	24.1	*0.12*	15.3
DG 34:2	Sample 1	*1.45*	2.7	*1.48*	6.4
	Sample 2	*3.37*	16.2	*3.53*	13.4
	Sample 3	*4.32*	6.3	*4.43*	7.0
	Sample 4	*7.55*	1.6	*4.79*	4.5
	Sample 5	*1.81*	2.6	*1.75*	6.4
DG 34:1	Sample 1	*1.87*	3.6	*1.92*	7.2
	Sample 2	*0.67*	15.1	*0.69*	14.8
	Sample 3	*1.85*	7.2	*1.89*	7.7
	Sample 4	*5.66*	1.9	*5.16*	2.1
	Sample 5	*0.36*	30.5	*0.33*	13.9
DG 36:5	Sample 1	*0.29*	6.6	*0.29*	10.3
	Sample 2	*1.09*	16.4	*1.14*	13.2
	Sample 3	*1.69*	5.7	*1.74*	8.2
	Sample 4	*0.05*	21.0	*0.05*	43.1
	Sample 5	*0.40*	4.5	*0.38*	7.2
DG 36:4	Sample 1	*4.32*	3.2	*4.38*	7.5
	Sample 2	*10.99*	16.2	*11.50*	13.2
	Sample 3	*14.31*	6.3	*14.68*	7.3
	Sample 4	*13.20*	2.3	*11.92*	4.0
	Sample 5	*5.95*	9.6	*5.90*	9.3
DG 36:3	Sample 1	*5.89*	2.3	*5.98*	6.1
	Sample 2	*5.82*	14.0	*6.04*	11.6
	Sample 3	*11.29*	6.9	*11.61*	8.1
	Sample 4	*43.50*	2.0	*39.27*	3.9
	Sample 5	*2.89*	4.6	*2.75*	5.6
DG 36:2	Sample 1	*10.27*	2.2	*10.43*	6.2
	Sample 2	*2.38*	14.4	*2.48*	11.7
	Sample 3	*6.50*	7.7	*6.65*	7.9
	Sample 4	*53.49*	2.2	*48.53*	3.2
	Sample 5	*1.9*	3.2	*1.83*	5.5

**Table 3:** Coefficient of variation (CV) of intra- and inter-day precision of TG species determined in five different human fecal samples by FIA-FTMS/MS analyzed in fivefold

Triacylglycerols		Intra-day	CV (%)	Inter-day	CV (%)
	Sample	Mean (*n* = 5) (nmol/mg dw)		Mean (*n* = 5) (nmol/mg dw)	
TG 50:2	Sample 1	*0.07*	5.4	*0.07*	8.9
	Sample 2	*0.13*	10.5	*0.14*	15.7
	Sample 3	*4.15*	7.1	*4.15*	7.4
	Sample 4	*3.50*	10.4	*3.17*	9.9
	Sample 5	*0.19*	97.3	*0.11*	118.3
TG 50:1	Sample 1	*0.17*	1.9	*0.18*	12.1
	Sample 2	*0.07*	18.5	*0.07*	20.4
	Sample 3	*1.26*	5.8	*1.26*	5.9
	Sample 4	*1.01*	10.5	*0.92*	9.9
	Sample 5	*0.06*	69.9	*0.04*	82.1
TG 50:0	Sample 1	*0.22*	10.5	*0.24*	20.1
	Sample 2	*0.05*	14.9	*0.05*	14.1
	Sample 3	n.d.	n.d.	n.d.	-
	Sample 4	n.d.	n.d.	n.d.	-
	Sample 5	*0.10*	7.4	*0.10*	9.9
TG 52:5	Sample 1	n.d.	n.d.	n.d.	-
	Sample 2	*0.11*	7.3	*0.11*	10.7
	Sample 3	*3.41*	7.8	*3.49*	11.9
	Sample 4	*2.10*	8.9	*1.91*	8.5
	Sample 5	n.d.	n.d.	n.d.	-
TG 52:4	Sample 1	*0.32*	5.9	*0.32*	14.2
	Sample 2	*0.82*	7.6	*0.84*	11.6
	Sample 3	*27.90*	6.9	*27.76*	7.8
	Sample 4	*20.26*	9.4	*18.36*	9.0
	Sample 5	*1.25*	87.4	*0.66*	116.8
TG 52:3	Sample 1	*0.39*	9.0	*0.41*	16.2
	Sample 2	*0.44*	13.5	*0.46*	13.4
	Sample 3	*18.36*	5.1	*18.30*	5.9
	Sample 4	*65.61*	10.4	*59.35*	10.1
	Sample 5	*0.35*	23.6	*0.37*	140.5
TG 52:2	Sample 1	*1.04*	11.1	*1.09*	14.8
	Sample 2	*0.21*	10.5	*0.22*	13.5
	Sample 3	*9.74*	4.9	*9.77*	6.0
	Sample 4	*45.75*	10.8	*41.29*	10.5
	Sample 5	*0.35*	92.8	*0.18*	129.3
TG 54:7	Sample 1	n.d.	n.d.	n.d.	-
	Sample 2	*0.24*	7.1	*0.24*	9.8
	Sample 3	*14.67*	8.6	*15.10*	14.7
	Sample 4	*0.21*	13.9	*0.19*	13.4
	Sample 5	n.d.	n.d.	n.d.	-
TG 54:6	Sample 1	*0.35*	6.0	*0.38*	28.8
	Sample 2	*1.24*	9.7	*1.27*	11.0
	Sample 3	*51.78*	5.6	*51.45*	6.7
	Sample 4	*20.86*	9.2	*18.89*	8.9
	Sample 5	*1.40*	74.4	*0.77*	104.8
TG 54:5	Sample 1	*0.82*	9.5	*0.85*	18.9
	Sample 2	*1.41*	10.9	*1.45*	10.9
	Sample 3	*52.48*	4.1	*52.47*	5.6
	Sample 4	*106.95*	10.4	*96.83*	10.0
	Sample 5	*1.85*	102.5	*0.92*	146.0
TG 54:4	Sample 1	*1.83*	11.6	*1.89*	15.3
	Sample 2	*0.93*	11.0	*0.96*	11.6
	Sample 3	*37.41*	4.1	*37.39*	5.7
	Sample 4	*175.39*	10.5	*146.61*	26.1
	Sample 5	*0.65*	28.9	*0.66*	147.5
TG 54:3	Sample 1	*4.78*	11.3	*4.91*	14.2
	Sample 2	*0.44*	12.0	*0.46*	12.8
	Sample 3	*18.69*	4.2	*18.72*	5.8
	Sample 4	*248.23*	12.4	*225.33*	11.5
	Sample 5	*0.6*	88.8	*0.29*	131.0

### Limit of quantification

Higher CV values were most likely related to concentrations close to limit of detection. Therefore, limits of quantification (LoQs) were determined functionally as described previously [21, 33]. Non-endogenous DG and TG species were spiked at various concentrations and analyzed in 5-fold. CV and accuracy were fitted as shown in Fig. S3 (see ESM). The calculated LoQs were in the range of 0.01–0.2 nmol/mg dw for DG species and 0.01–0.3 nmol/mg dw for TG species. LoQs determined at CV of 20% were significantly lower compared with those determined by accuracy. Most of the LoQs derived from CVs were in the range of 0.01 to 0.02 nmol/mg dw which also matched the inter- and intra-day CVs listed in Tables 2 and 3. This demonstrates a reproducible analysis below 0.1 nmol/mg dw. LoQs derived from accuracy analysis depend on accurate addition of low amounts of DG/TG species, which may be compromised by many factors including analyte absorption or inhomogeneity issues (described below). Except a poor curve fit as a factor, we could not find an explanation for the order of magnitude difference between the LoQs determined for different species. There seems to be neither a relation to species chain length nor number of double bonds. LoQs for DG and TG appear to be similar. Based on these considerations, we applied for practical reasons 0.02 nmol/mg dw as LoD and 0.1 nmol/mg dw as LoQ (this is also substantiated by data from dilution integrity testing shown below).

### Recovery, linearity, and dilution integrity

Recovery of DG and TG species was determined at two spike levels (ESM Table S1). Most of the determined recoveries were within the expected range of 85 to 115%. However, considering the high complexity of fecal material as matrix, we think that recoveries between 75 and 135% are acceptable.

To further evaluate the dynamic range of the method, the linearity of quantification was tested for several species not present in fecal samples. DG 36:6, DG 38:0, TG 54:2, and TG 54:1 were spiked at six different concentrations (ESM Fig. S4). All species revealed a good correlation of spiked and detected concentrations. However, species response seems to depend on structural features, as described for cholesteryl ester [26], and should be studied in detail in further studies. A linear range covering most of the tested samples was demonstrated up to 120 mg dw/mL and 90 mg dw/mL for DG and TG, respectively.

Moreover, dilution integrity was tested by quantification of gradually diluted stool samples (1.6 to 0.02 mg dw/mL). Low (DG 32:0, TG 48:0), medium (DG 34:2, TG 52:2), and high (DG 36:3, TG 54:4) abundant species showed a good correlation of expected and measured concentrations (ESM Fig. S5). The assay was linear at low (DG 32:0 and TG 48:0) and high concentrations (DG 36:3, TG 54:4) and matched the above described LoQ and LoD and linear range (up to 250 mg DG dw/mL and 150 mg TG dw/mL), respectively.

### Evaluation of reproducibility issues

As described above, very high variations were observed for some samples (about 10% of tested fecal samples). Therefore, various experiments were performed to evaluate the origin of irreproducibility. Despite thorough mechanical homogenization, fecal samples are suspensions and a lack of homogeneity may cause variations. Therefore, we first tested whether centrifugation affects DG and TG concentrations. Five samples showing high variations were analyzed without centrifugation as well as after centrifugation (ESM Table S2). While DG species were detected in both pellet and supernatant, TG species were found in three of the samples enriched in the pellet. However, the DG/TG species profiles of supernatant and pellet closely resembled each other (ESM Table S3), suggesting that centrifugation does not separate a specific pool of these lipid classes.

In order to improve the homogeneity, we tested addition of detergent (0.1%, 0.5%, and 1.0% SDS), an additional homogenization step using the Precellys^®^ homogenizer (data not shown) as well as sonication up to 3 h (ESM Table S4). All of these additional treatments did not result in a decrease of variation. In contrast, the application of higher sample volumes (2 mg instead of 200 μg dw) for lipid extraction showed some decrease of CV, especially for TG species and samples with a high fraction of TG in the pellet. Considering that variation is mainly due to sample inhomogeneity, drawing a higher sample volume could explain lower variations.

In a next step, we asked whether these inhomogeneity issues could be related to the consistency of the fecal material. Therefore, we selected, if available, three samples for each stool grade (according to Bristol Stool Chart [34]; with grade 1 representing hard and grade 7 watery consistency) from a study on fiber and polyunsaturated fatty acid interventions [22]. The samples were measured in five replicates (Table 4). Samples with grades 3 to 5 showed CVs ≤ 10%. However, in samples with lower grades (1 to 2), we could not see a clear trend for higher CVs, which may have been expected for more solid consistency.

**Table 4:** DG and TG concentrations and their coefficient of variation (*n* = 5) related to stool grading

		Diacylglycerol		Triacylglycerol	
	sample	Mean (*n* = 5) (nmol/mg dw)	CV (%)	Mean (*n* = 5) (nmol/mg dw)	CV (%)
Grade 1	Sample a	*46.11*	6.6	*2.31*	11.6
	Sample b	*15.71*	7.6	*14.25*	6.1
	Sample c	*54.27*	6.4	*8.55*	10.7
Grade 2	Sample d	*78.73*	7.1	*10.69*	10.9
	Sample e	*38.65*	26.3	*82.10*	27.6
	Sample f	*11.62*	2.1	*1.11*	20.8
Grade 3	Sample g	*93.86*	9.2	*38.27*	10.2
	Sample h	*27.30*	6.0	*2.65*	7.5
	Sample i	*29.44*	6.5	*39.34*	5.1
Grade 4	Sample j	*52.35*	3.0	*5.15*	7.9
	Sample k	*14.30*	5.8	*1.87*	6.5
	Sample l	*21.41*	2.7	*2.15*	2.5
Grade 5	Sample m	*66.36*	6.4	*15.67*	6.0
	Sample n	*25.91*	8.9	*19.96*	8.0
	Sample o	*94.64*	4.2	*11.83*	5.5
Grade 6	Sample p	*65.84*	4.8	*10.28*	5.6
	Sample q	*44.92*	3.8	*21.40*	5.2
	Sample r	*49.10*	17.0	*39.42*	15.2
Grade 7	Sample s	*46.94*	22.2	*1.74*	18.6

**Fig. 2:**
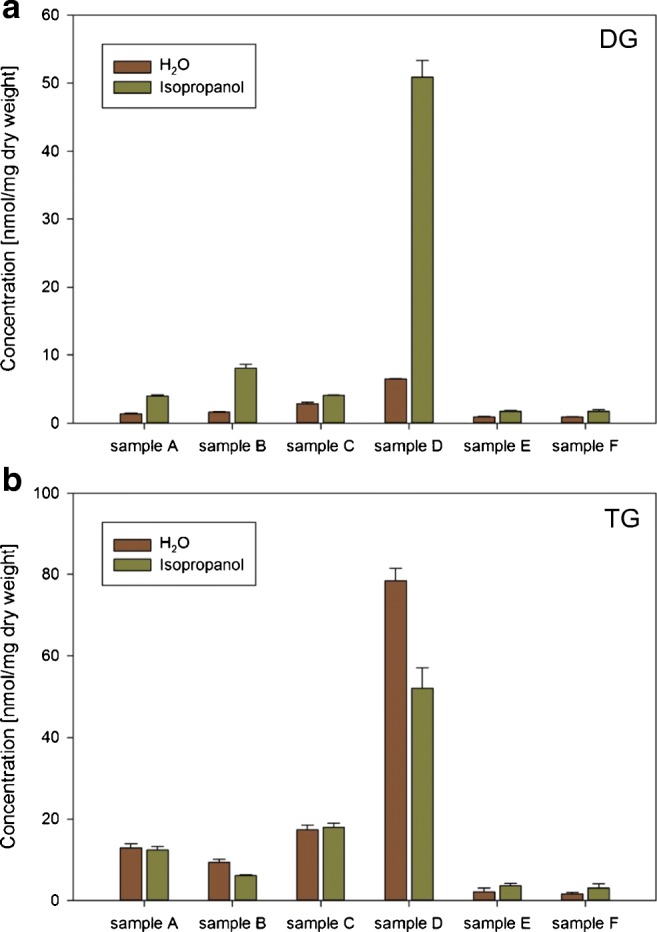
Effect of isopropanol addition on DG (**a**) and TG (**b**) quantification. Displayed are six individual samples homogenized in water and supplemented with the same volume of either H_2_O (brown) or isopropanol (green) (70% related to volume)

Finally, we checked whether the solvent used for sample preparation may affect DG/TG concentrations. In several studies, homogenization of fecal material was performed not only in water [35] or aqueous buffer [36, 37], but also in diluted organic solvents [38, 39]. In our laboratory, diluted isopropanol was used to stabilize fecal concentrations of short chain fatty acids [40]; thus, the effect of isopropanol was investigated for DG/TG concentrations. Therefore, fecal raw material was homogenized in water and subsequently diluted at ratios of 3 to 7 (by volume) with either water or isopropanol (Fig. 2) and immediately stored at − 80 °C. Unexpectedly, addition of isopropanol tremendously increased DG concentrations in almost all samples. Moreover, in two of the six samples, we observed a drop of TG concentrations. However, the increase of DG could not be explained by TG degradation in these samples because the increase of DG exceeded the decreased amount of TG. Comparison of spectra of samples stabilized in water or isopropanol showed clear differences in all DG species profiles and in the TG profiles of three of the six samples (ESM Table S5). We could not observe additional species upon isopropanol addition and no common pattern in the increased DG species for the individual samples.

**Fig. 3:**
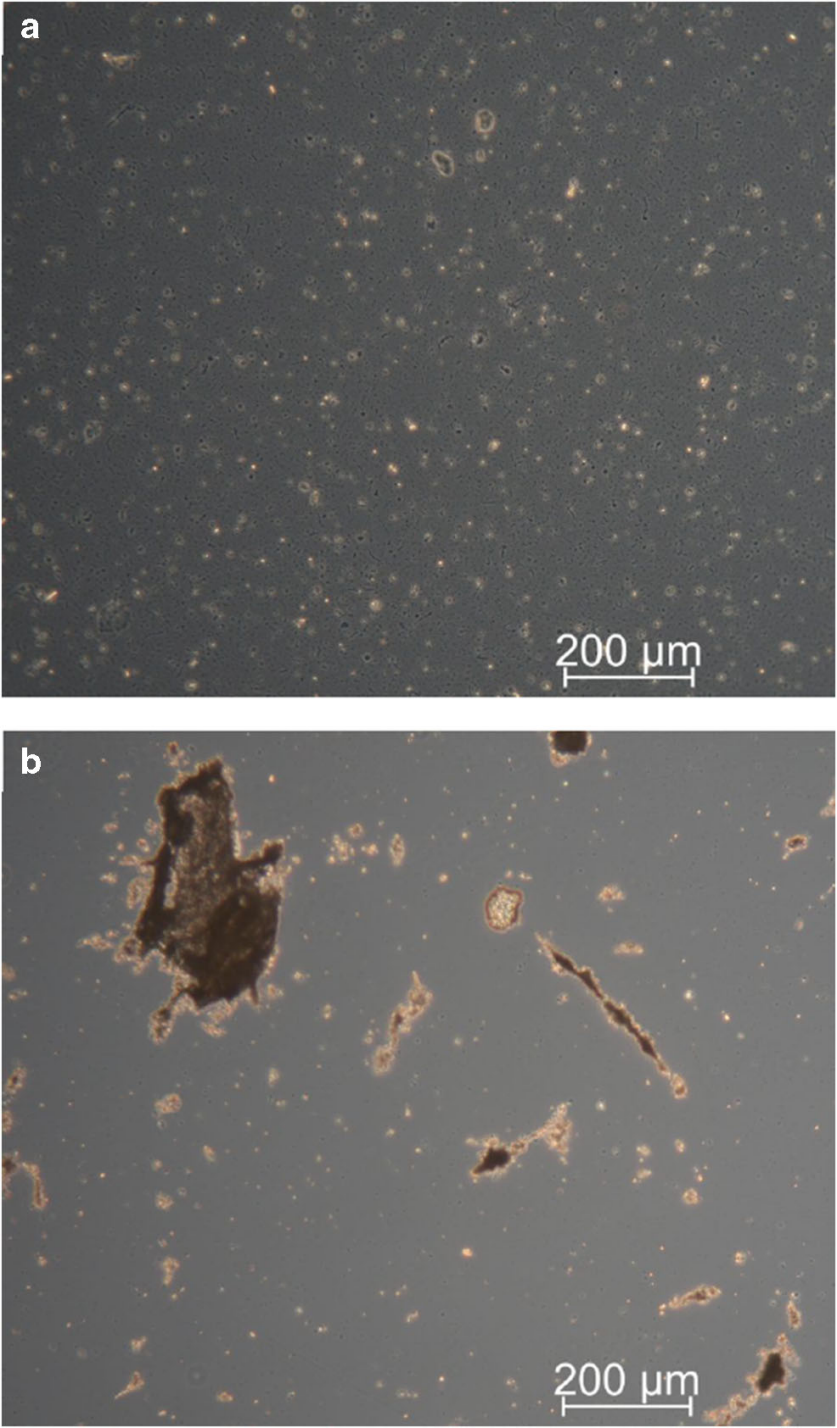
Comparison of human fecal sample D diluted either in water (**a**) or in isopropanol (**b**) at a dry weight of 2 mg dw/mL using phase-contrast microscopy with × 10 magnification

To get more insight, we examined both aqueous and isopropanol-containing sample homogenates by light microscopy (Fig. 3). Clearly, aqueous samples seem to be more homogeneous compared with isopropanol-containing samples. However, in aqueous samples, a massive presence of bacteria could be observed. To inhibit metabolic activity and to reduce health risks, fecal samples are frequently treated with alcohols. Currently, we cannot explain the aggregation induced by addition of isopropanol. The increase of DG upon isopropanol addition seems to be related to both disruption of bacteria resulting in improved extractability of DG and lipolysis of TG. The latter seems to be triggered in some samples by addition of isopropanol and matches lipolytic activities observed in organic solvents [31, 32]. These data clearly demonstrate that further studies are warranted to evaluate optimal pre-analytic conditions for fecal samples as well as the origin of these differences.

## Conclusion

Here we report, to our knowledge, the first method for the identification and quantification of DG and TG in human fecal material using FIA coupled to a high-resolution FTMS instrument. Up to now, only a few studies on the fecal lipidome exist which is most likely related to the difficulties faced with this sample material [2, 16]. The proposed method has a short run time of 4 min per sample, including MS2 measurements, facilitating a high sample throughput necessary for clinical studies. Validation of the novel method demonstrated its suitability for large-scale studies despite the higher variations observed for some samples. These variations are related to inhomogeneity of samples and lipolytic activity that requires further investigations considering pre-analytical issues as an essential part of lipidomic workflows and their standardization [29, 30, 41]. Therefore, we recommend performing measurements in triplicate, when high accuracy is needed. In this regard, sampling is very important since metabolites are distributed highly heterogeneous in feces and homogenization of larger quantities is recommended [42].

In summary, the presented method provides a valuable tool to quantify DG and TG species, the major lipid classes in human fecal samples. These data could be a first step to unravel the fecal lipidome and get more insight into its role for health and disease.

## Electronic supplementary material

ESM 1 (PDF 799 kb)
