# Supplementary material for: Quantification of diacylglycerol and triacylglycerol species in human fecal samples by flow injection Fourier transform mass spectrometry
Source: Anal Bioanal Chem. 2020 Mar 21;412(10):2315–26. doi: 10.1007/s00216-020-02416-y (PMC7118049; doi:10.1007/s00216-020-02416-y)
Supplement: Supplementary file 1 — (PDF 799 kb) [file 216_2020_2416_MOESM1_ESM.pdf]

Analytical and Bioanalytical Chemistry

Electronic Supplementary Material

Quantification of diacylglycerol and triacylglycerol species in human fecal samples by flow injection Fourier transform mass spectrometry

Verena M. Ertl, Marcus Höring, Hans-Frieder Schött, Christina Blücher, Louise Kjølbæk, Arne Astrup, Ralph Burkhardt, Gerhard Liebisch

Content

- Figure S1: IS mass spectra
- Figure S2: MS2-spectra
- Figure S3: Limit of quantification
- Figure S4: Linearity
- Figure S5: Dilution integrity
- Table S1: Recovery
- Table S2: Effect of centrifugation on DG/TG concentration
- Table S3: Effect of centrifugation on DG/TG species profiles
- Table S4: Effect of sonication on DG/TG concentration
- Table S5: Effect of solvent on DG/TG concentration

A

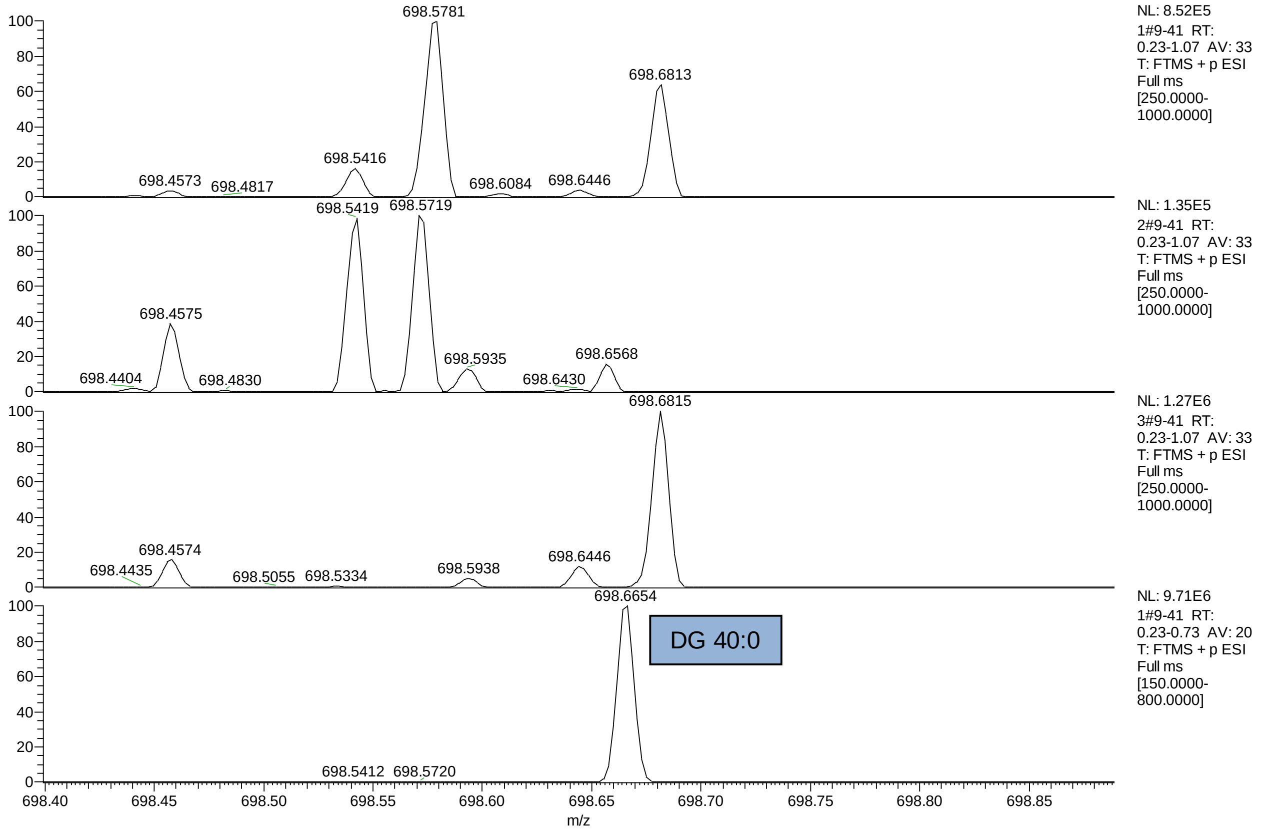

698.5781
698.6813
698.4573
698.4817
698.5416
698.6084
698.6446
NL: 8.52E5
1#9-41 RT: 0.23-1.07 AV: 33
T: FTMS + p ESI Full ms [250.0000-1000.0000]
698.5419
698.5719
698.4575
698.4404
698.4830
698.5935
698.6430
698.6568
NL: 1.35E5
2#9-41 RT: 0.23-1.07 AV: 33
T: FTMS + p ESI Full ms [250.0000-1000.0000]
698.6815
698.4574
698.4435
698.5055
698.5334
698.5938
698.6446
NL: 1.27E6
3#9-41 RT: 0.23-1.07 AV: 33
T: FTMS + p ESI Full ms [250.0000-1000.0000]
698.6654
DG 40:0
698.5412
698.5720
NL: 9.71E6
1#9-41 RT: 0.23-0.73 AV: 20
T: FTMS + p ESI Full ms [150.0000-800.0000]
m/z

B

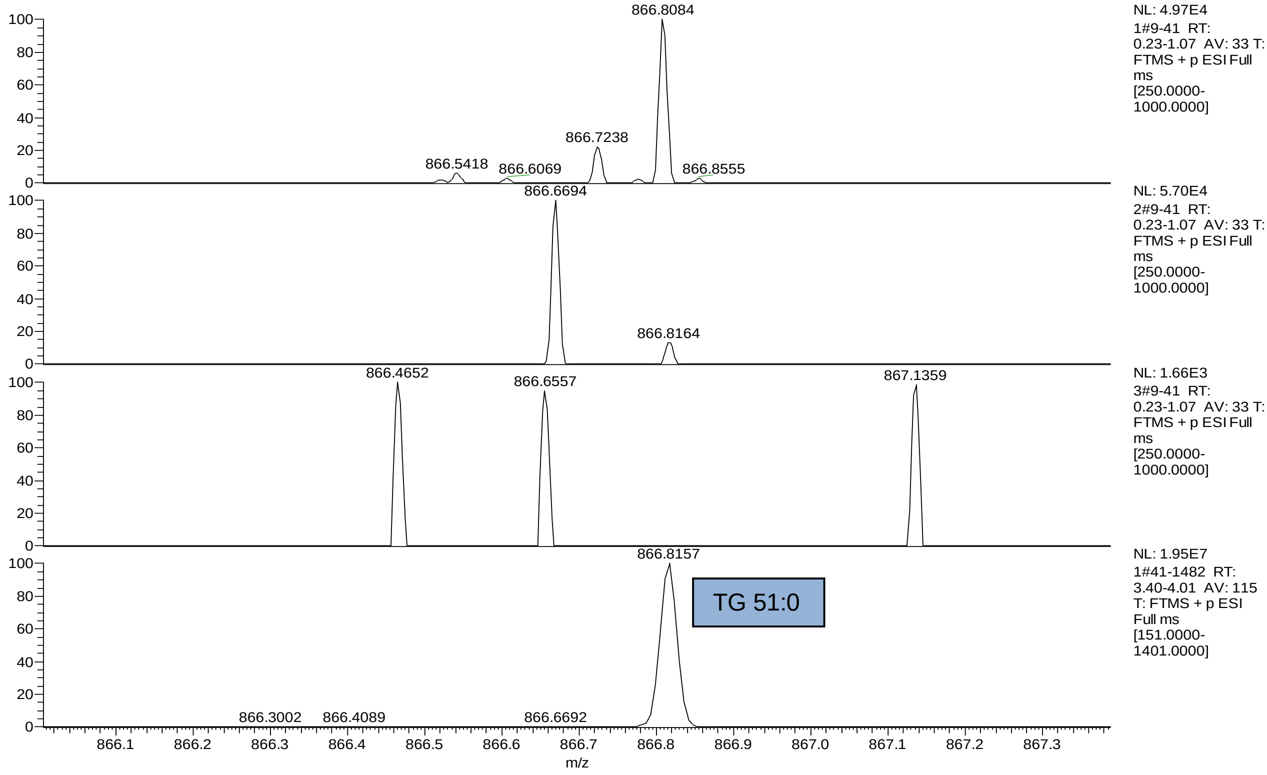

866.8084
866.7238
866.5418
866.6069
866.8555
NL: 4.97E4
1#9-41 RT: 0.23-1.07 AV: 33 T: FTMS + p ESI Full ms [250.0000-1000.0000]
866.6694
866.8164
NL: 5.70E4
2#9-41 RT: 0.23-1.07 AV: 33 T: FTMS + p ESI Full ms [250.0000-1000.0000]
866.4652
866.6557
867.1359
NL: 1.66E3
3#9-41 RT: 0.23-1.07 AV: 33 T: FTMS + p ESI Full ms [250.0000-1000.0000]
866.8157
TG 51:0
866.3002
866.4089
866.6692
NL: 1.95E7
1#41-1482 RT: 3.40-4.01 AV: 115
T: FTMS + p ESI Full ms [151.0000-1401.0000]
m/z

C

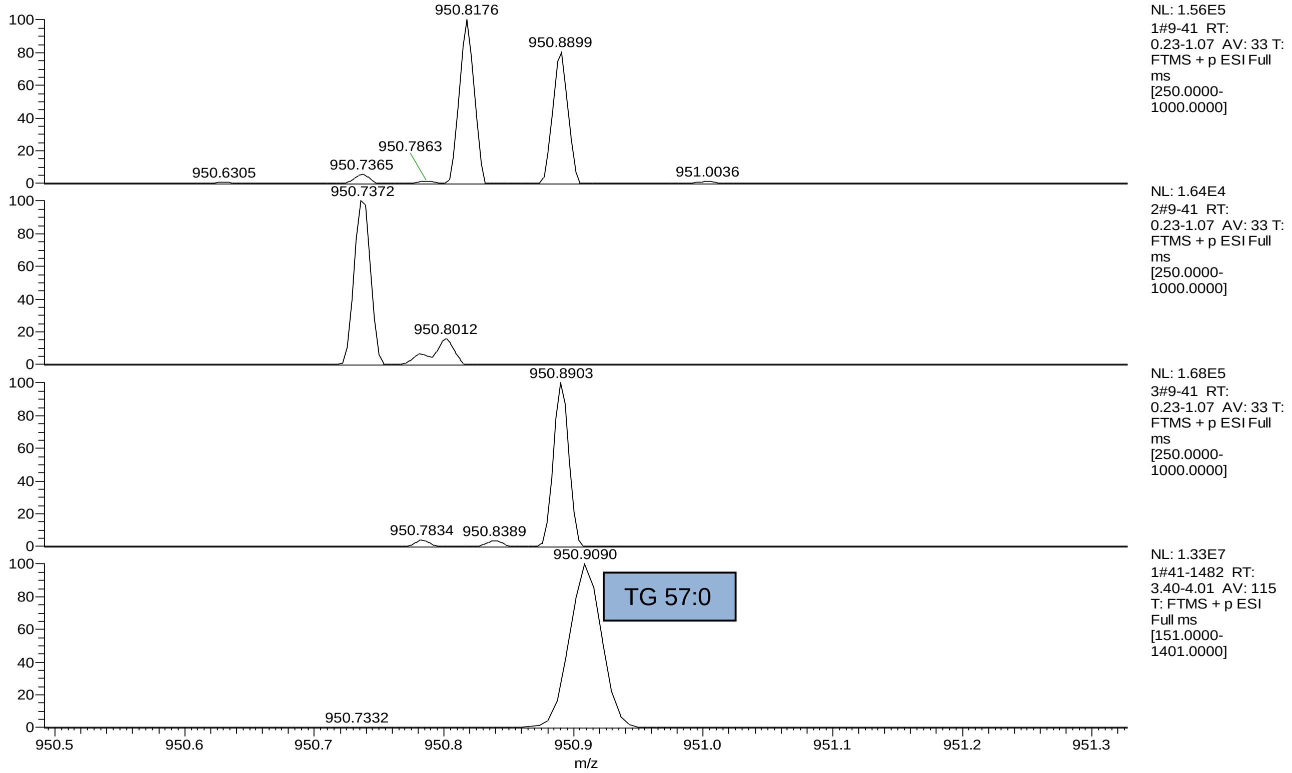

Fig. S1 Displayed are zoomed mass spectra from four individual human fecal samples analyzed in positive ion mode. Panel A shows the mass range of DG 40:0 (*m/z* 698.665701), panel B of TG 51:0 (*m/z* 866.817117) and panel C of TG (*m/z* 950.911017) for samples without (upper three spectra) and with applied internal standards (bottom spectrum)

DG 34:2 → DG 16:0_18:2

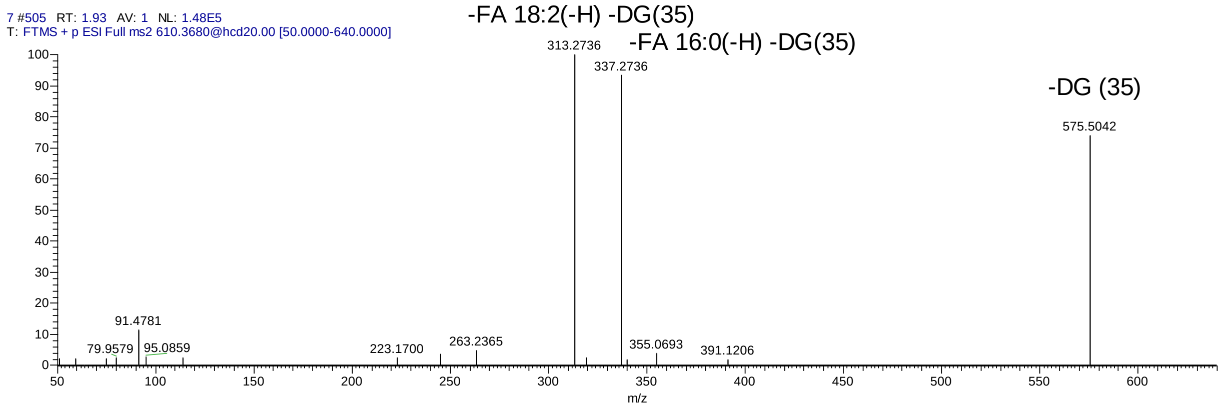

DG 36:4 → DG 18:2_18:2

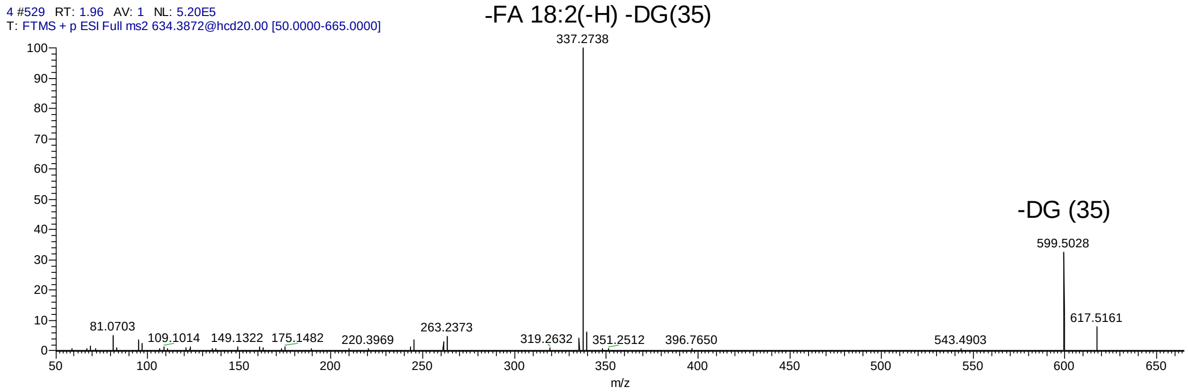

DG 36:3 → DG 18:1_18:2

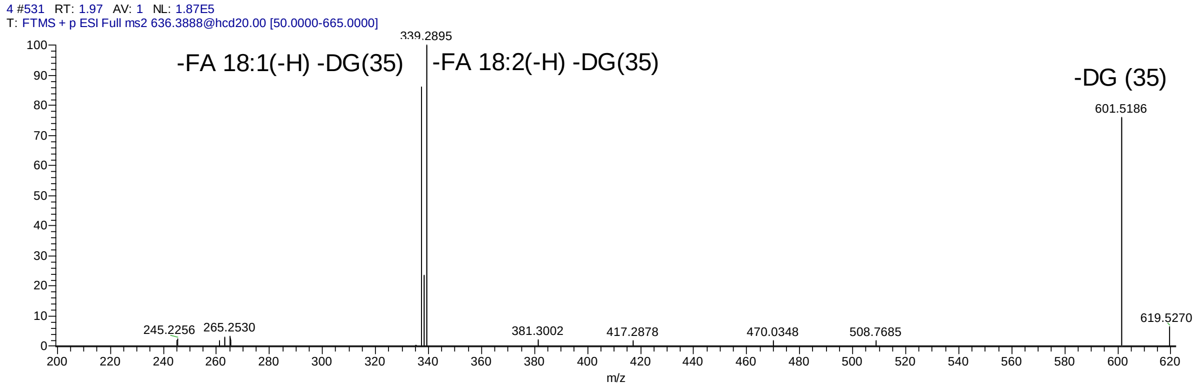

DG 36:2 → DG 18:0_18:2 ; DG 18:1_18:1

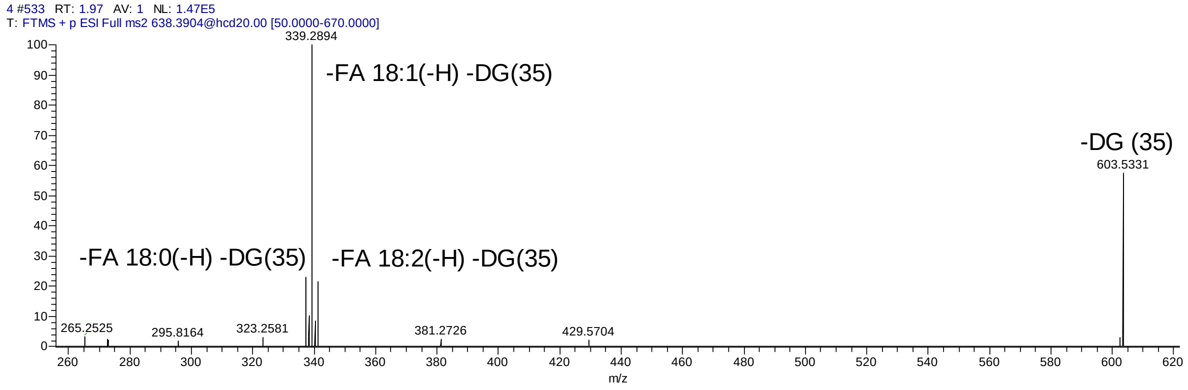

DG 30:0 → DG 12:0_18:0

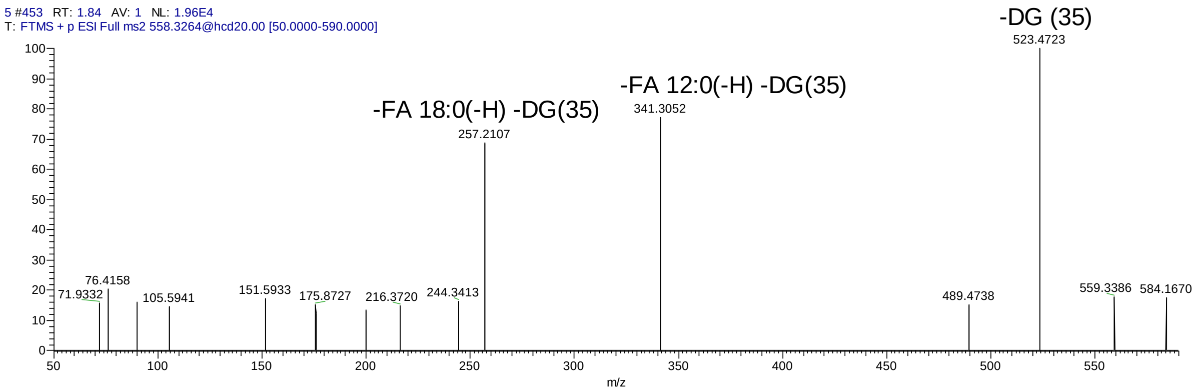

TG 50:0 → TG 16:0_16:0_18:0

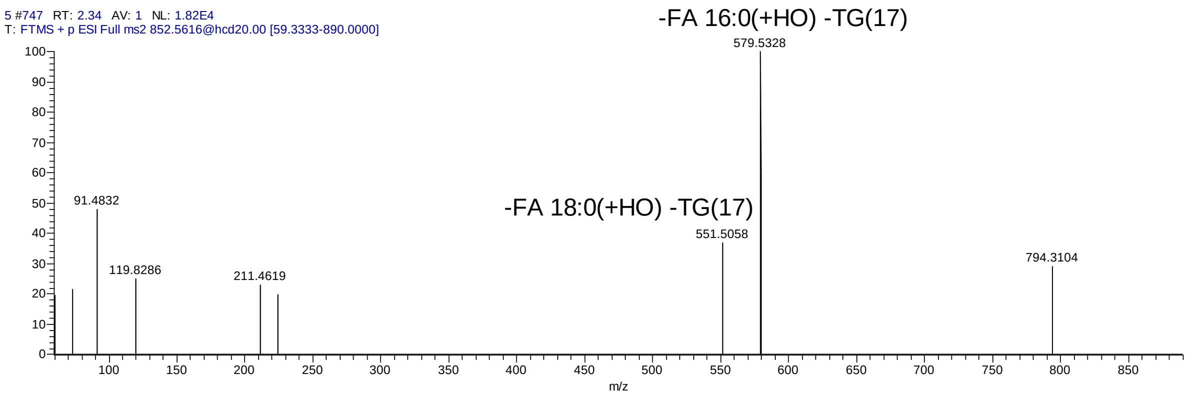

TG 52:4 → TG 16:0_18:2_18:2

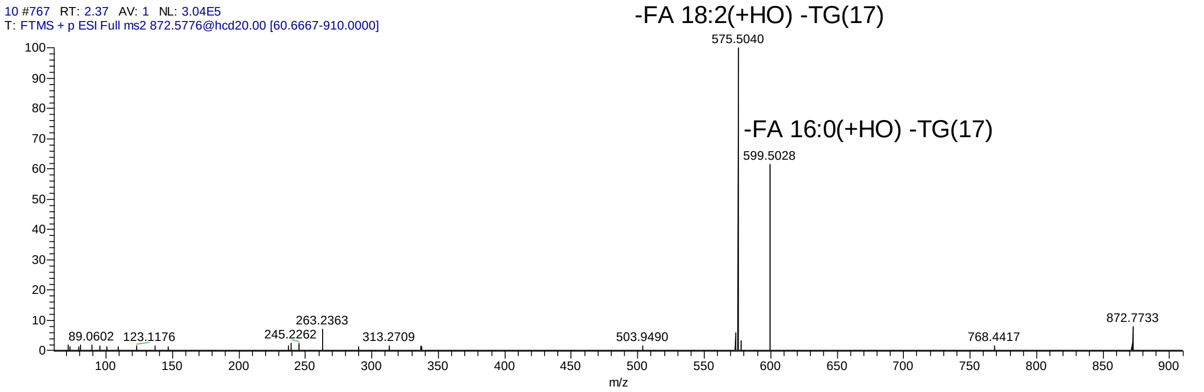

TG 54:6 → TG 18:0_18:3_18:3 ; TG 18:1_18:2_18:3 ; TG 18:2_18:2_18:2

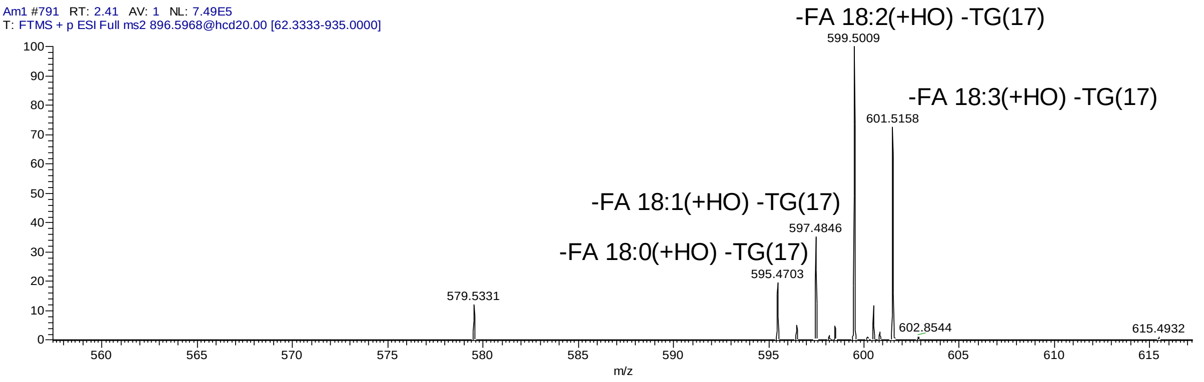

TG 54:5 → TG 18:1_18:2_18:2

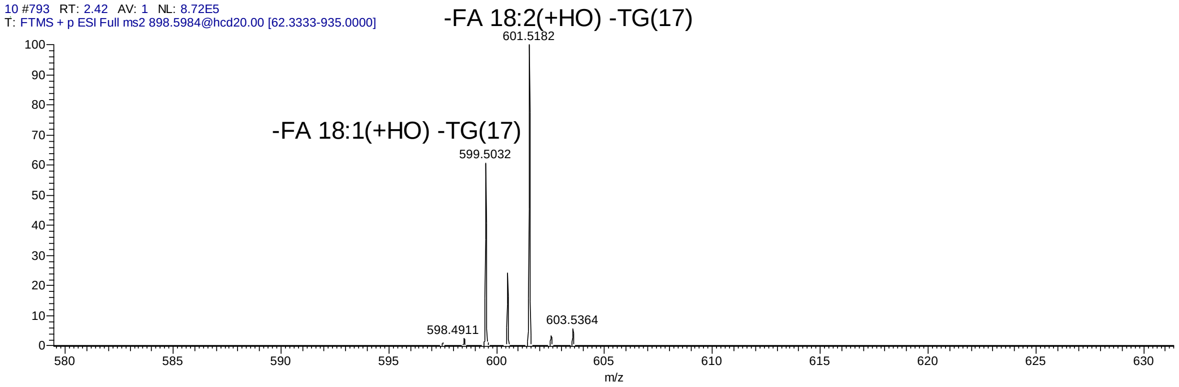

TG 54:4 → TG 18:1_18:1_18:2

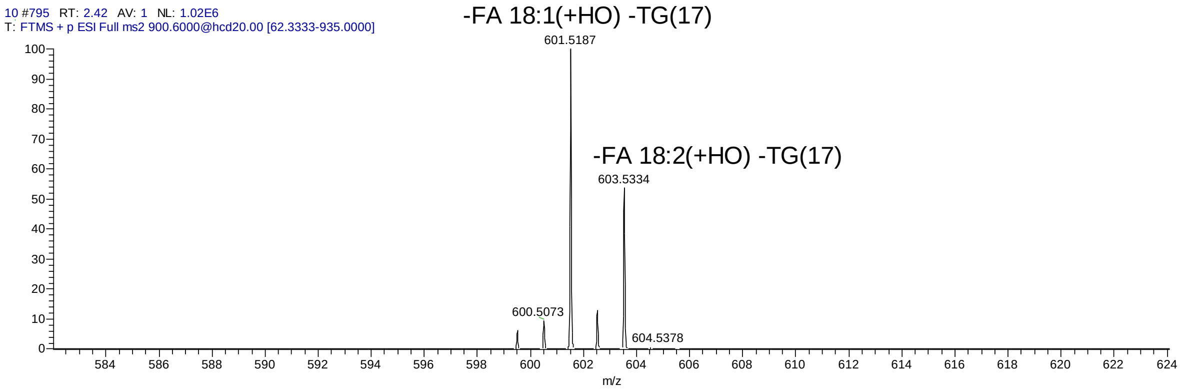

TG 54:3 → TG 18:1_18:1_18:1

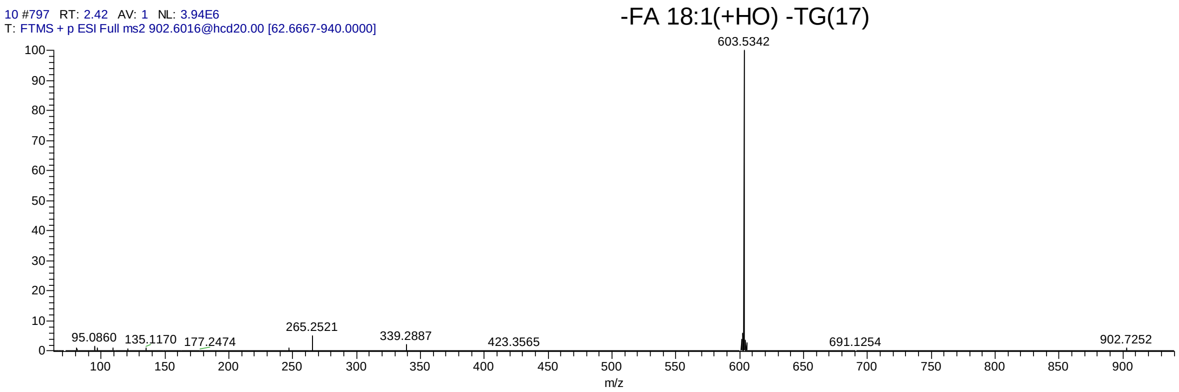

Fig. S2 MS2 spectra of DG and TG species and acyl combinations derived from the spectra

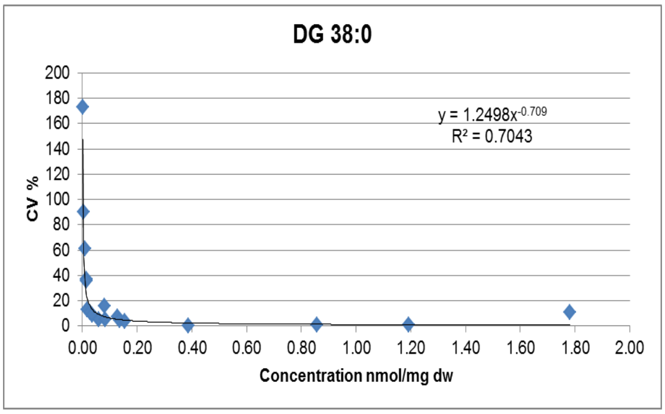

LoQ at CV = 20%: 0.02 nmol/mg dw

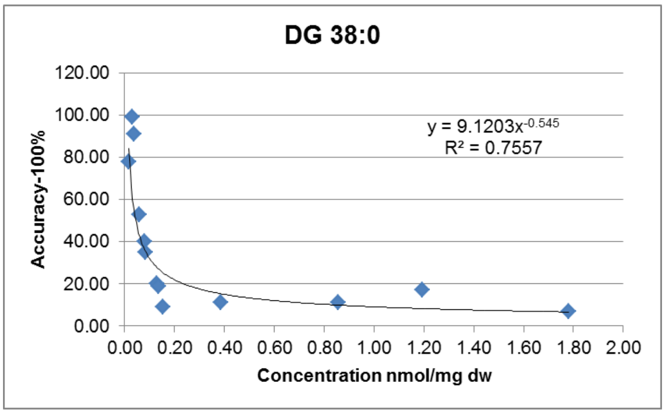

LoQ at trueness-100 = 20%: 0.2 nmol/mg dw

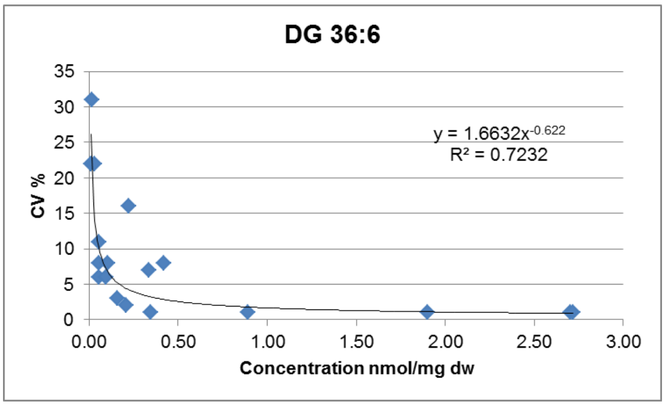

LoQ at CV = 20%: 0.02 nmol/mg dw

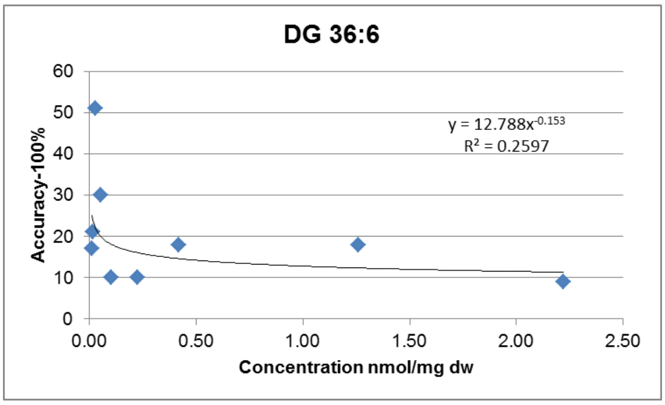

LoQ at trueness-100 = 20%: 0.05 nmol/mg dw

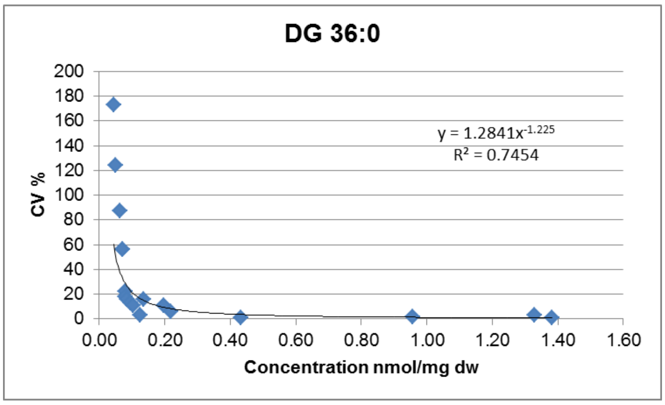

LoQ at CV = 20%: 0.1 nmol/mg dw

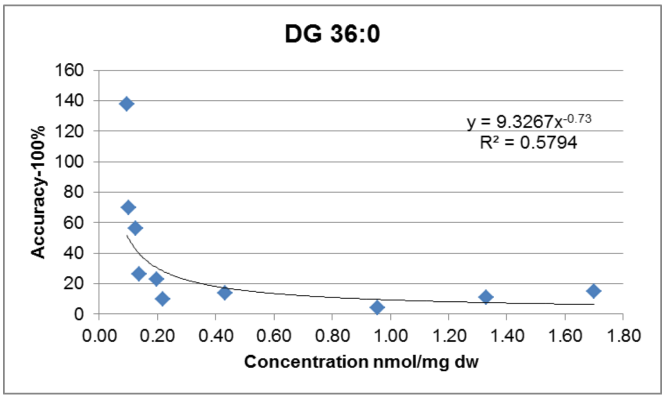

LoQ at trueness-100 = 20%: 0.3 nmol/mg dw

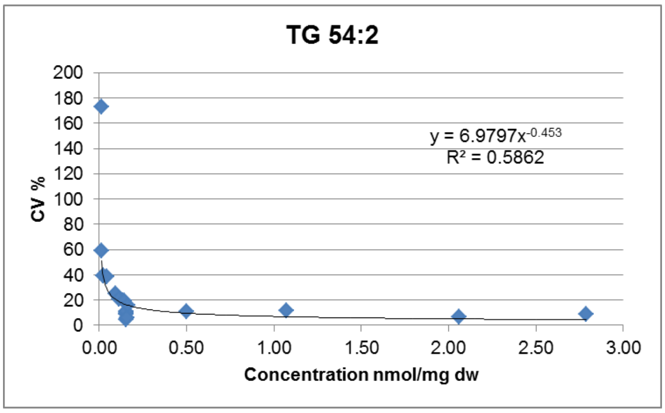

LoQ at CV = 20%: 0.1 nmol/mg dw

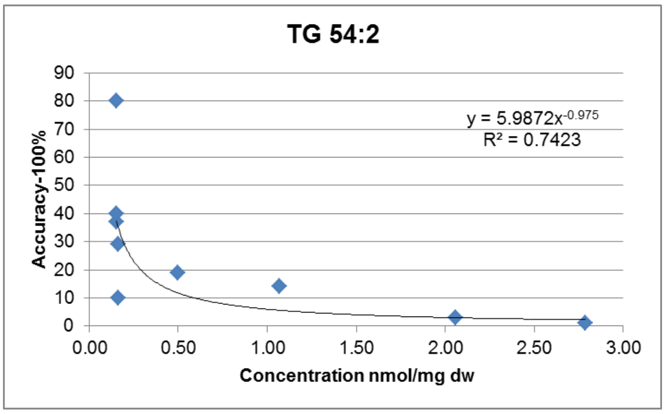

LoQ at trueness-100 = 20%: 0.3 nmol/mg dw

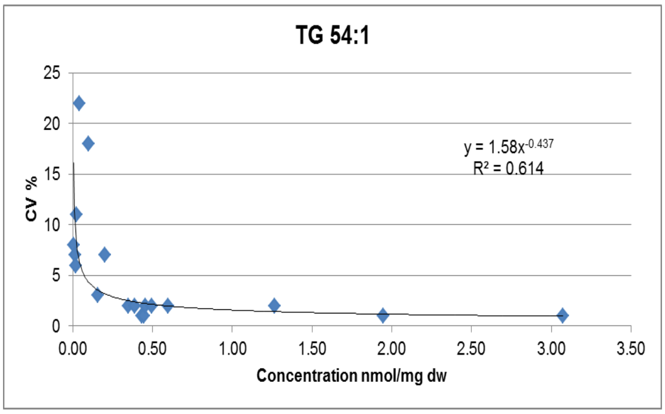

LoQ at CV = 20%: 0.01 nmol/mg dw

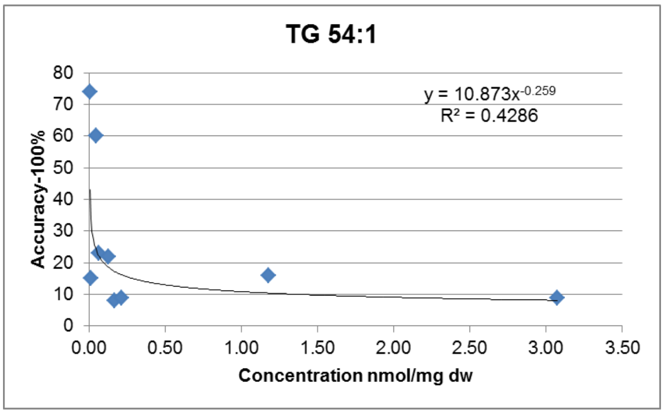

LoQ at trueness-100 = 20%: 0.09 nmol/mg dw

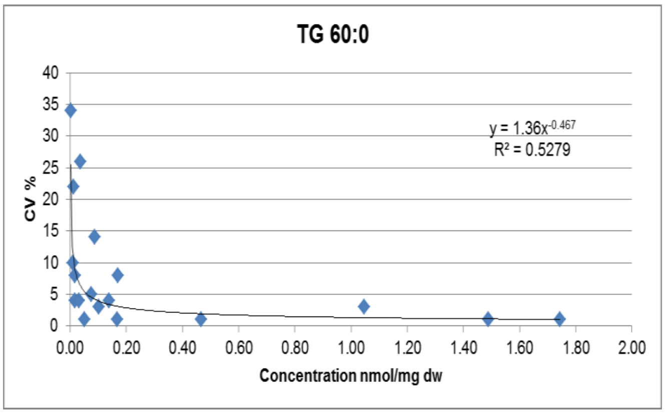

LoQ at CV = 20%: 0.01 nmol/mg dw

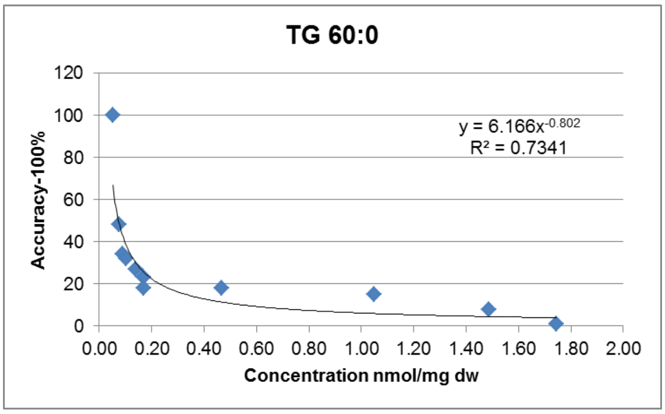

LoQ at trueness-100 = 20%: 0.2 nmol/mg dw

Fig. S3 Calculation of LoQ for DG 38:0, DG 36:6, DG 36:0, TG 54:2, TG 54:1 and TG 60:0 from serial dilutions of different human fecal samples each analyzed in fivefold. The left panel illustrates the measured CVs plotted against the concentration of undiluted samples. The right panel shows the absolute values of trueness-100 plotted against the concentration of undiluted samples. The results were fitted by a power function and concentrations were calculated at CV = 20% or at absolute values of trueness-100 = 20%

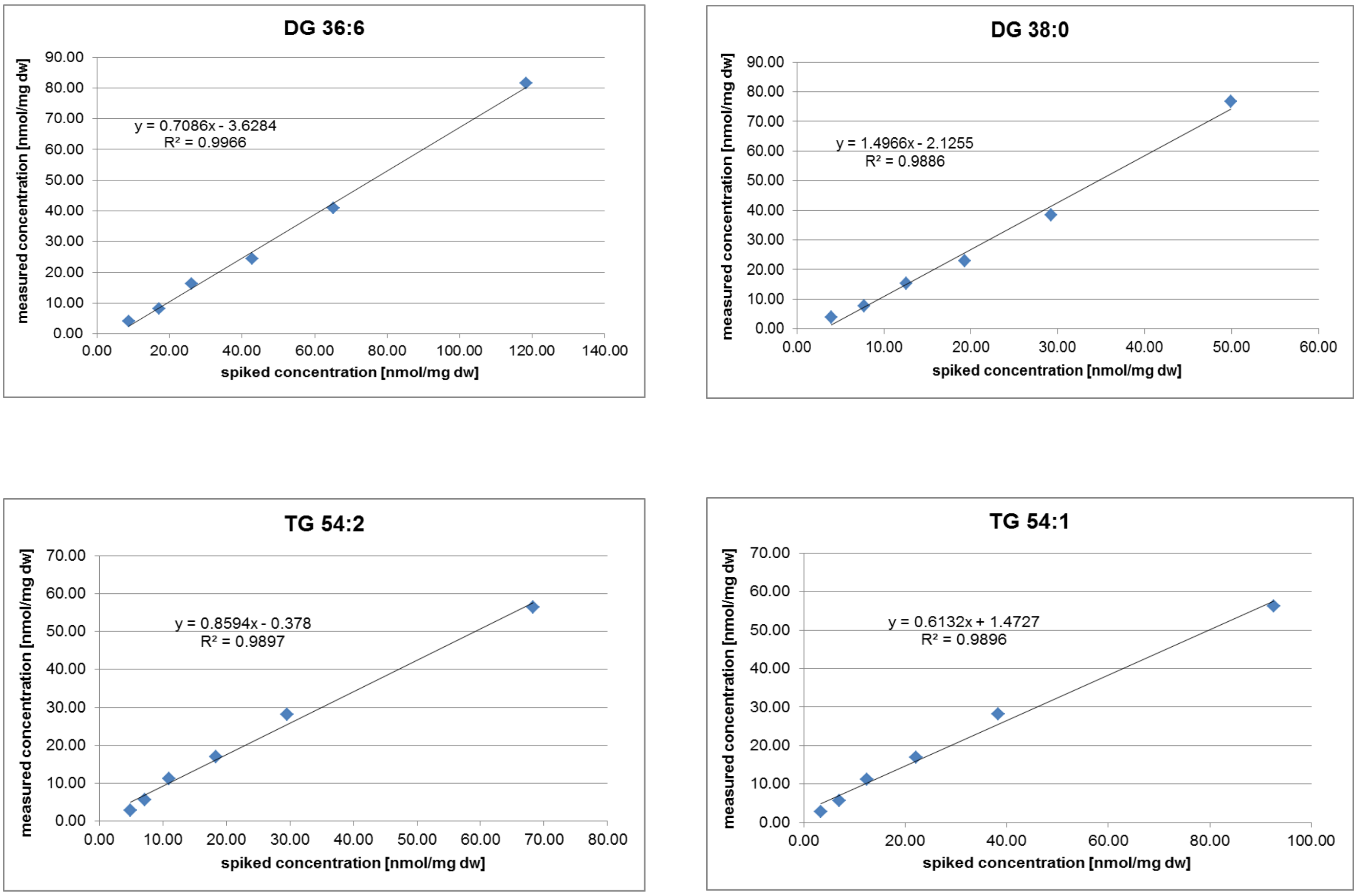

Fig. S4 Linearity of DG and TG standards. Displayed are means (n=5) of the measured plotted against the spiked concentrations

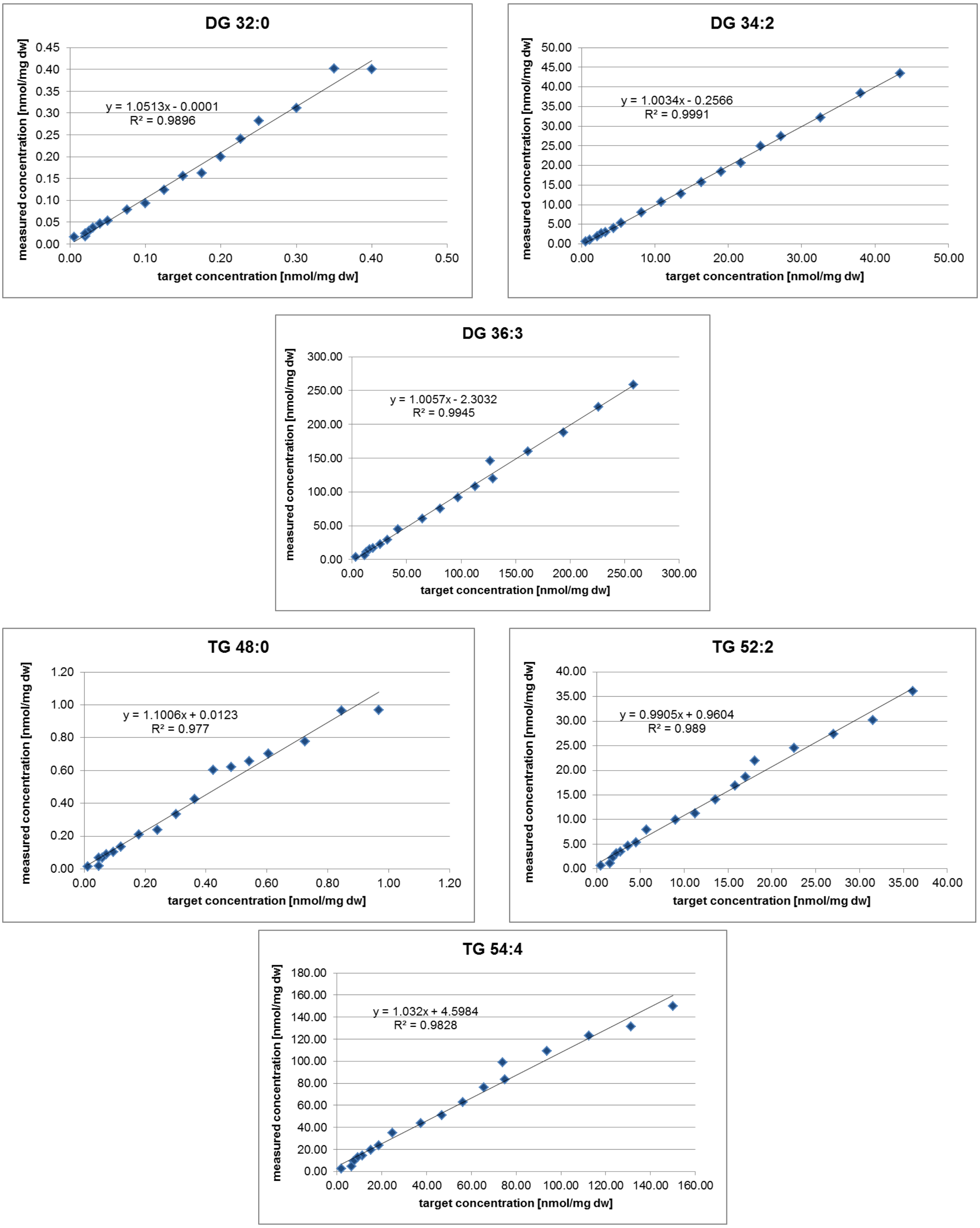

Fig. S5 Dilution integrity of DG and TG species with low, medium, and high concentrations, respectively. Samples were analyzed in triplicates. The mean measured concentrations were plotted against the target concentrations

Table S1 Recovery data of DG and TG species in human feces. Concentrations were determined in triplicates

**Compound**		Spiked concentration [nmol/mg dw]	Concentration ± standard deviation [nmol/mg dw]			Recovery [%]
**DG 36:6**	unspiked		0.04	±	0.02	
	Spike low	3.27	4.33	±	0.21	132.6
	Spike high	16.3	17.3	±	0.95	105.9
**DG 36:4**	unspiked		0.99	±	0.03	
	Spike low	3.24	3.93	±	0.13	121.1
	Spike high	16.2	16.2	±	0.86	99.8
**DG 36:0**	unspiked		0.07	±	0.02	
	Spike low	3.20	2.72	±	0.09	84.8
	Spike high	16.0	12.1	±	0.42	75.3
**DG 38:0**	unspiked		0.01	±	0.01	
	Spike low	3.07	3.13	±	0.06	102.0
	Spike high	15.3	14.8	±	0.47	96.8
**TG 48:0**	unspiked		0.13	±	0.05	
	Spike low	2.48	2.04	±	0.12	82.1
	Spike high	12.4	10.4	±	0.44	84.3
**TG 54:6**	unspiked		0.03	±	0.01	
	Spike low	2.28	2.52	±	0.10	110.9
	Spike high	11.4	12.7	±	0.89	111.6
**TG 54:3**	unspiked		0.02	±	0.01	
	Spike low	2.26	2.04	±	0.08	90.2
	Spike high	11.3	10.2	±	0.65	90.5
**TG 54:1**	unspiked		0.02	±	0.01	
	Spike low	2.25	2.32	±	0.05	103.3
	Spike high	11.3	11.8	±	0.54	104.7

Table S2a Five different samples were analyzed without centrifugation as well as their supernatant and pellet after centrifugation. Each sample was analyzed in triplicate. Displayed are mean DG species concentrations and the fraction found in supernatant and pellet, respectively

Diglyceride	sample	without centrifugation [nmol/mg dw]	supernatant [%]	pellet [%]
**DG 34:3**	sample 1	0.19	46.5	53.5
	sample 2	0.13	53.0	47.0
	sample 3	0.07	53.1	46.9
	sample 4	0.13	57.9	42.1
	sample 5	0.06	41.7	58.3
**DG 34:2**	sample 1	2.97	47.9	52.1
	sample 2	1.62	48.3	51.7
	sample 3	0.56	46.4	53.6
	sample 4	1.15	53.4	46.6
	sample 5	1.16	44.2	55.8
**DG 34:1**	sample 1	0.78	48.0	52.0
	sample 2	6.73	48.3	51.7
	sample 3	2.07	45.6	54.4
	sample 4	1.48	53.4	46.6
	sample 5	0.20	46.6	53.4
**DG 36:5**	sample 1	0.20	47.8	52.2
	sample 2	0.10	50.3	49.7
	sample 3	0.05	59.7	40.3
	sample 4	0.24	55.0	45.0
	sample 5	0.25	45.2	54.8
**DG 36:4**	sample 1	13.35	47.9	52.1
	sample 2	3.15	50.0	50.0
	sample 3	1.19	51.4	48.6
	sample 4	3.66	55.4	44.6
	sample 5	3.81	45.1	54.9
**DG 36:3**	sample 1	5.85	48.1	51.9
	sample 2	12.21	48.5	51.5
	sample 3	3.42	47.2	52.8
	sample 4	4.43	53.8	46.2
	sample 5	1.88	45.6	54.4
**DG 36:2**	sample 1	2.79	47.6	52.4
	sample 2	47.41	48.0	52.0
	sample 3	13.67	45.7	54.3
	sample 4	7.25	53.1	46.9
	sample 5	1.25	46.1	53.9

Table S2b Five different samples were analyzed without centrifugation as well as their supernatant and pellet after centrifugation. Each sample was analyzed in triplicate. Displayed are mean TG species concentrations and the fraction found in supernatant and pellet, respectively

Triglyceride	sample	without centrifugation [nmol/mg dw]	supernatant [%]	pellet [%]
**TG 52:4**	sample 1	0.26	47.8	52.2
	sample 2	0.24	22.0	78.0
	sample 3	0.18	20.7	79.3
	sample 4	0.13	44.0	56.0
	sample 5	0.13	11.2	88.8
**TG 52:3**	sample 1	0.08	45.8	54.2
	sample 2	0.75	27.1	72.9
	sample 3	0.73	11.7	88.3
	sample 4	0.22	38.4	61.6
	sample 5	0.04	8.3	91.7
**TG 52:2**	sample 1	0.04	40.5	59.5
	sample 2	2.65	25.7	74.3
	sample 3	2.68	9.7	90.3
	sample 4	0.63	37.2	62.8
	sample 5	0.02	8.4	91.6
**TG 54:7**	sample 1	0.01	44.1	55.9
	sample 2	0.02	17.2	82.8
	sample 3	0.01	15.4	84.6
	sample 4	0.02	48.2	51.8
	sample 5	0.02	11.8	88.2
**TG 54:6**	sample 1	0.46	47.8	52.2
	sample 2	0.39	17.1	82.9
	sample 3	0.21	21.9	78.1
	sample 4	0.17	44.3	55.7
	sample 5	0.15	8.7	91.3
**TG 54:5**	sample 1	0.31	47.8	52.2
	sample 2	1.12	30.2	69.8
	sample 3	0.92	14.2	85.8
	sample 4	0.42	38.7	61.3
	sample 5	0.09	9.1	90.9
**TG 54:4**	sample 1	0.20	47.3	52.7
	sample 2	4.29	27.7	72.3
	sample 3	3.73	10.6	89.4
	sample 4	1.06	35.7	64.3
	sample 5	0.06	9.1	90.9
**TG 54:3**	sample 1	0.07	47.6	52.4
	sample 2	14.66	27.5	72.5
	sample 3	13.59	8.7	91.3

sample 4	2.99	38.2	61.8
sample 5	0.03	12.3	87.7

Species	sample 1 without centrifug [%]	sample 1 supernatant [%]	sample 1 pellet [%]	sample 2 without centrifug [%]	sample 2 supernatant [%]	sample 2 pellet [%]	sample 3 without centrifug [%]	sample 3 supernatant [%]	sample 3 pellet [%]	sample 4 without centrifug [%]	sample 4 supernatant [%]	sample 4 pellet [%]	sample 5 without centrifug [%]	sample 5 supernatant [%]	sample 5 pellet [%]
DG 34:3	0.7	0.4	0.5	0.2	0.2	0.1	0.3	0.3	0.2	0.7	0.6	0.5	0.7	0.6	0.7
DG 34:2	11.2	11.5	11.6	2.2	2.2	2.2	2.5	2.6	2.6	5.9	6.0	6.0	12.7	12.5	13.1
DG 34:1	2.9	2.0	2.0	9.1	9.2	9.2	9.3	9.3	9.5	7.6	7.3	7.5	2.2	2.2	2.1
DG 36:5	0.7	0.6	0.6	0.1	0.1	0.1	0.2	0.2	0.1	1.2	1.2	1.1	2.7	2.7	2.7
DG 36:4	50.1	49.0	49.6	4.3	4.2	3.9	5.4	5.3	4.3	18.8	18.3	17.2	41.5	41.5	42.0
DG 36:3	22.0	23.2	23.3	16.6	16.6	16.4	15.5	16.0	15.4	22.8	22.9	23.0	20.5	20.2	20.1
DG 36:2	10.5	10.7	11.0	64.4	64.6	65.0	61.8	61.5	62.8	37.3	38.1	39.2	13.7	13.8	13.5

Table S3a Species profile in % total DG of the data listed in Table S2a

Species	sample 1 without centrifug [%]	sample 1 supernatant [%]	sample 1 pellet [%]	sample 2 without centrifug [%]	sample 2 supernatant [%]	sample 2 pellet [%]	sample 3 without centrifug [%]	sample 3 supernatant [%]	sample 3 pellet [%]	sample 4 without centrifug [%]	sample 4 supernatant [%]	sample 4 pellet [%]	sample 5 without centrifug [%]	sample 5 supernatant [%]	sample 5 pellet [%]
TG 52:4	18.0	19.3	19.0	1.0	1.0	1.3	0.8	1.6	0.7	2.3	3.0	2.4	24.0	24.5	20.4
TG 52:3	5.7	5.3	5.7	3.1	3.2	3.2	3.3	3.8	3.0	4.0	4.3	4.2	6.9	7.0	8.1
TG 52:2	3.0	2.3	3.0	11.0	10.7	11.5	12.2	12.0	11.9	11.2	11.4	11.8	4.0	3.1	3.6
TG 54:7	0.9	0.9	1.0	0.1	0.1	0.1	0.0	0.1	0.0	0.3	0.4	0.3	3.8	3.9	3.1
TG 54:6	31.7	32.3	31.7	1.6	1.5	2.8	0.9	1.7	0.7	3.0	3.7	2.8	28.1	26.9	29.7
TG 54:5	21.8	21.6	21.2	4.7	4.9	4.2	4.2	5.5	3.5	7.5	8.3	8.1	17.2	17.8	18.9
TG 54:4	14.1	13.8	13.8	17.8	18.2	17.6	16.9	18.1	16.3	18.7	17.9	19.8	10.9	11.3	12.0
TG 54:3	4.9	4.5	4.5	60.8	60.5	59.4	61.6	57.3	63.9	53.0	51.0	50.6	5.1	5.5	4.1

Table S3b Species profile in % total TG of the data listed in Table S2b

		without ultrasonication				ultrasonication			
		Diglyceride		Triglyceride		Diglyceride		Triglyceride	
	sample	Mean (n=3) [nmol/mg dw]	CV [%]	Mean (n=3) [nmol/mg dw]	CV [%]	Mean (n=3) [nmol/mg dw]	CV [%]	Mean (n=3) [nmol/mg dw]	CV [%]
**10 minutes**	sample I	**4.08**	8.3	**0.13**	11.7	**4.14**	9.9	**0.22**	89.4
	sample II	**48.69**	10.6	**4.74**	15.7	**46.91**	2.3	**4.87**	13.1
	sample II	**9.08**	6.9	**1.33**	19.8	**8.86**	4.2	**1.25**	11.0
**30 minutes**	sample I	**4.22**	4.6	**0.13**	10.2	**4.52**	1.1	**0.20**	51.8
	sample II	**50.73**	6.8	**4.99**	2.1	**46.93**	4.3	**5.06**	0.6
	sample II	**9.29**	5.7	**1.33**	10.5	**9.76**	4.1	**1.35**	13.8
**60 minutes**	sample I	**4.35**	1.9	**0.12**	5.2	**4.39**	6.3	**0.12**	1.8
	sample II	**48.85**	4.2	**5.04**	16.3	**50.15**	3.6	**5.00**	3.0
	sample II	**8.89**	3.2	**1.20**	5.2	**9.88**	3.1	**1.53**	33.0
**120 minutes**	sample I	**4.39**	2.6	**0.18**	55.9	**4.34**	3.2	**0.12**	3.8
	sample II	**49.79**	10.8	**4.67**	7.1	**47.36**	7.2	**4.65**	7.6
	sample II	**8.89**	1.5	**1.16**	2.9	**9.42**	4.0	**1.18**	2.8
**180 minutes**	sample I	**4.19**	1.7	**0.12**	5.0	**6.97**	64.0	**0.59**	136.0
	sample II	**48.89**	3.6	**6.18**	34.6	**49.91**	5.7	**4.55**	4.7
	sample II	**9.15**	8.7	**1.11**	5.2	**10.59**	6.4	**1.29**	17.2

Table S4 Calculation of CV for DG and TG species either with or without ultrasonication during extraction. Incubation time varies from 10 to 180 minutes. Each sample was analyzed in three replicates

Species	sample A		sample B		sample C		sample D		sample E		sample F	
	H2O [%]	Isopropanol [%]	H2O [%]	Isopropanol [%]	H2O [%]	Isopropanol [%]	H2O [%]	Isopropanol [%]	H2O [%]	Isopropanol [%]	H2O [%]	Isopropanol [%]
DG 34:3	0.6	0.6	0.0	0.4	1.0	0.9	1.3	0.9	2.1	1.6	8.7	5.3
DG 34:2	7.1	5.9	19.2	12.8	10.8	8.6	6.1	7.2	8.4	8.2	10.0	10.7
DG 34:1	8.6	7.0	9.5	5.7	13.5	11.8	4.5	1.9	8.1	7.8	19.1	15.0
DG 36:5	1.3	1.4	1.1	1.7	2.5	2.3	3.7	2.1	3.9	7.9	9.1	4.5
DG 36:4	13.3	13.0	23.5	27.1	18.3	16.3	24.4	43.6	26.5	20.9	16.2	22.9
DG 36:3	26.3	24.6	21.8	25.6	16.3	15.5	29.4	33.0	26.4	20.7	14.4	18.5
DG 36:2	42.8	47.5	24.9	26.7	37.5	44.6	30.6	11.3	24.7	33.0	22.4	23.1

Table S5a DG species profile in % total DG for aqueous and isopropanol-containing samples (Figure 2)

Species	sample A		sample B		sample C		sample D		sample E		sample F	
	H2O [%]	Isopropanol [%]	H2O [%]	Isopropanol [%]	H2O [%]	Isopropanol [%]	H2O [%]	Isopropanol [%]	H2O [%]	Isopropanol [%]	H2O [%]	Isopropanol [%]
TG 52:4	3.5	3.8	14.1	14.4	5.0	4.9	5.2	8.4	6.2	9.3	4.5	7.5
TG 52:3	4.8	5.1	8.7	8.4	3.8	3.8	5.0	5.1	3.4	6.3	6.0	5.9
TG 52:2	9.8	10.0	7.2	7.2	12.1	12.2	4.3	2.1	10.2	6.9	15.4	11.9
TG 54:7	0.6	0.8	1.5	1.6	1.4	1.6	4.2	5.7	9.0	7.6	1.5	1.5
TG 54:6	5.0	5.1	17.1	16.7	7.1	7.4	11.8	22.0	7.5	14.0	8.3	13.8
TG 54:5	9.9	9.3	16.4	15.8	5.4	5.6	23.3	32.5	8.3	14.5	7.3	11.0
TG 54:4	23.0	22.5	14.0	13.6	13.0	12.9	23.4	18.0	11.8	14.6	14.2	14.0
TG 54:3	43.5	43.4	20.9	22.4	52.1	51.6	22.7	6.3	43.6	26.8	42.9	34.3

Table S5b DG species profile in % total TG for aqueous and isopropanol-containing samples (Figure 2)
